# Misadventure of an Unsafe Abortion

**DOI:** 10.7759/cureus.31131

**Published:** 2022-11-05

**Authors:** Punit Hans, Kanchan Sharma

**Affiliations:** 1 Obstetrics and Gynecology, Patna Medical College, Patna, IND; 2 Obstetrics and Gynecology, Nalanda Medical College and Hospital, Patna, IND; 3 Obstetrics and Gynecology, Manipal Tata Medical College, Jamshedpur, IND

**Keywords:** systemic inflammatory response syndrome (sirs), sirs, uterine perforation, unsafe abortion, septic abortion, abortion

## Abstract

We are presenting a case of complicated unsafe abortion that landed in sepsis. A 21-year-old morbidly febrile but conscious female patient was brought on a stretcher by her attendants in the Gynecology emergency room. A foul fecal stench was coming from her body. On detailed and sympathetic questioning, the patient revealed she underwent an abortion by a local birth attendant in her village at 19 weeks of pregnancy. After that, she had pain abdomen, intermittent bleeding per vagina, difficulty in passing stool, loss of appetite, and fever. She took some local treatment but her condition gradually deteriorated and fecal-smelling vaginal discharge started. The patient was immediately shifted to the Intensive Care Unit. The decision for laparotomy was made by the team of gynecologists and surgeons as the patient's condition was not improving. During laparotomy, fetal parts present in the paracolic gutter were taken out. Post laparotomy, the patient was shifted to ICU, and her condition gradually improved then she was shifted to the recovery ward after five days.

## Introduction

An unsafe abortion is defined as an abortion performed by an unskilled or untrained provider using unsafe or less-studied methods to terminate a pregnancy [1]. Each year, worldwide 25 million unsafe abortions are reported [2]. In 2015, seven million complicated unsafe abortions occurred in low socioeconomic countries [3,4], and the mortality rate was also high about 220 deaths per 100,000 unsafe abortions [5]. There are many factors that increase the morbidity of unsafe abortion such as unsterile conditions, lack of antiseptic precautions, poor skill, poor instrumentations, and use of toxic substances. The infection rate is very high in an unsafe abortion landing the patient into sepsis. Here, we are presenting a case of complicated unsafe abortion that progressed to sepsis, leading to severe morbidity.

## Case presentation

A 21-year-old morbidly febrile but conscious female patient was brought on a stretcher by her attendants in our Gynecology emergency. A foul fecal stench was coming from her body. Attendants told the patient was alright seven days back but deteriorated after some operative treatment and was admitted to a local health center where conservative treatment was given. On detailed and sympathetic questioning, the patient revealed that she underwent an abortion by a local birth attendant in her village at 19 weeks of pregnancy. Following this, she had pain abdomen, intermittent bleeding per vagina, difficulty in passing stool, loss of appetite, and fever. She took treatment from local doctors but her condition gradually deteriorated and fecal-smelling vaginal discharge started. She came to know about her present pregnancy two months back after consulting a local doctor for infrequent menses. She took an abortifacient for the termination of her pregnancy which failed, resulting in the progression of the pregnancy. Regarding her menstrual history, she was not sure of her last menstrual period as her cycles were infrequent and irregular. As per her obstetric history, she was unmarried, para zero with one induced abortion of 19 weeks. No past medical investigation reports were presented by the patient or attendants.

On general examination, the patient was ill-looking and febrile with a temperature of 101ᵒF, severe pallor, dehydrated, and tachypneic with a respiratory rate of 31 breaths per minute, a pulse rate of 120 beats per minute and blood pressure was 98/60 mmHg. Her chest and cardiovascular examinations were normal. On per abdominal examination, distension with guarding, rigidity and tenderness were present. On pelvic examination fecal mucopurulent discharge was seen per speculum, cervical motion tenderness was positive, uterine size could not be assessed due to pain, and a boggy swelling was felt in the pouch of Douglas.

The patient was immediately shifted to the Intensive care unit. All routine investigations were sent, and serum lactate, coagulation profile, and complete metabolic panel were done. Blood and urine samples, vaginal swabs, and swabs from the cervix were sent for culture. Aggressive treatment in the line of systemic inflammatory response syndrome was started with higher antibiotics (Piperacillin + Tazobactum + Metronidazole), IV fluids, Foleys catheterization, and nasogastric tube insertion were started. Two units of packed red blood cells were arranged after proper grouping and cross-matching. Imaging studies with an x-ray abdomen erect view showed gas under the diaphragm and ultrasonography of the whole abdomen with pelvis showed an echogenic collection in both the uterine and pelvic cavities. Her investigation report showed marked leukocytosis of 18,000 per microliter (normal leukocyte count: 3,800-10,300 per microliter), C-reactive protein was 61 mg per liter ( normal value < 10 mg per liter), serum creatinine was 1.8 mg per deciliter (normal range for females: 0.6-1.1 mg per deciliter), hemoglobin was 7 g per deciliter (normal range:11.9 to 14.8 g/deciliter), ESR was high, liver function tests and serum electrolytes were within normal limits with slight hyponatremia (serum sodium - 133 milliequivalents per liter, normal range: 135-145 mEq/L) which was corrected with Normal saline infusion. The decision for laparotomy was made by the team of gynecologists and surgeons after two units of blood were arranged as the patient's condition was not improving.

**Figure 1:**
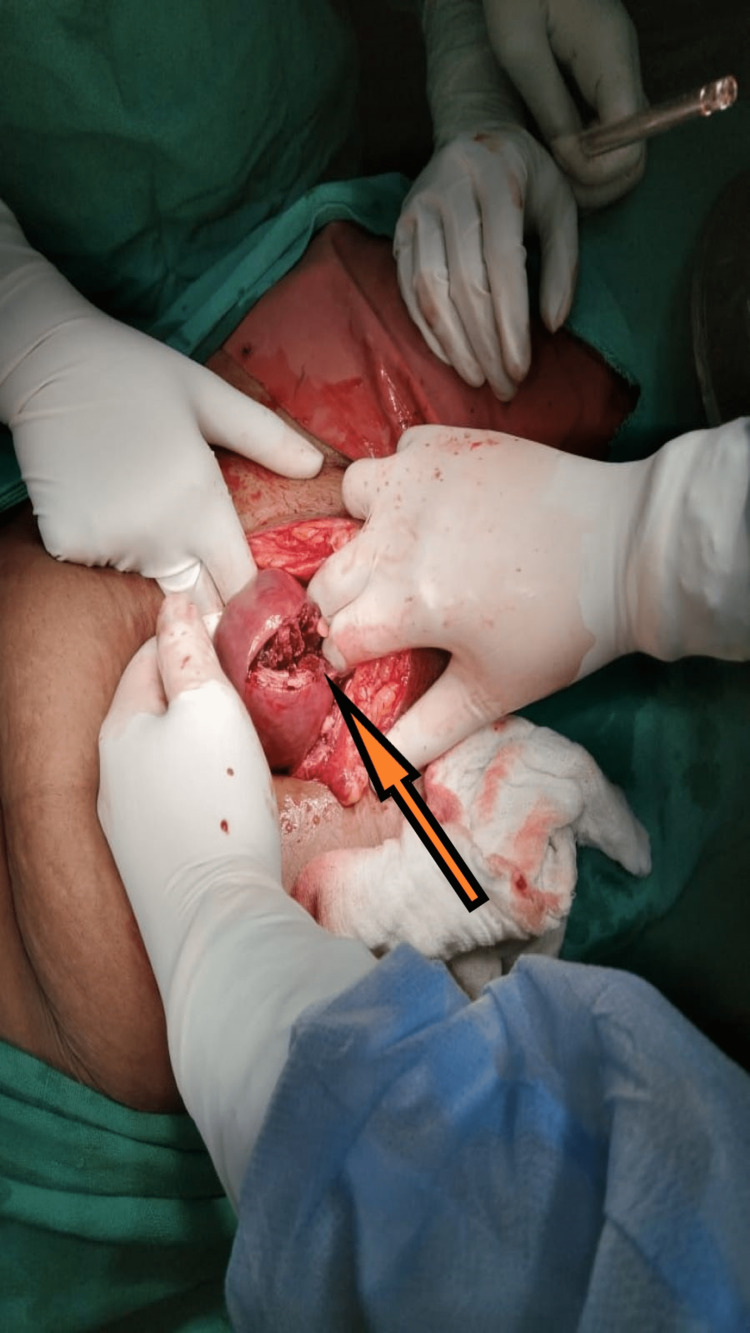
Intraoperative picture showing perforated uterus The arrow shows the site of the perforated uterus.

**Figure 2:**
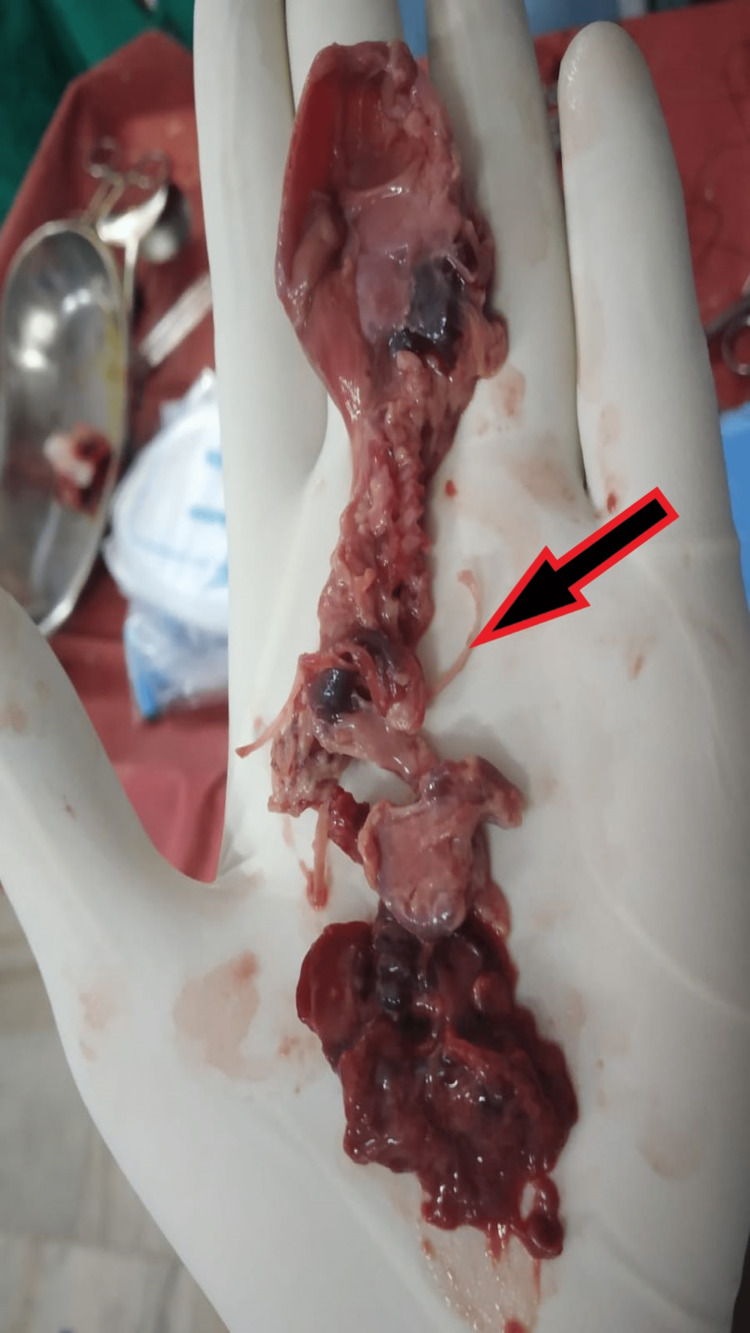
Intraoperative picture showing fetal parts and tissue taken out from para-colic gutter The arrow shows the fetal remnant parts.

**Figure 3:**
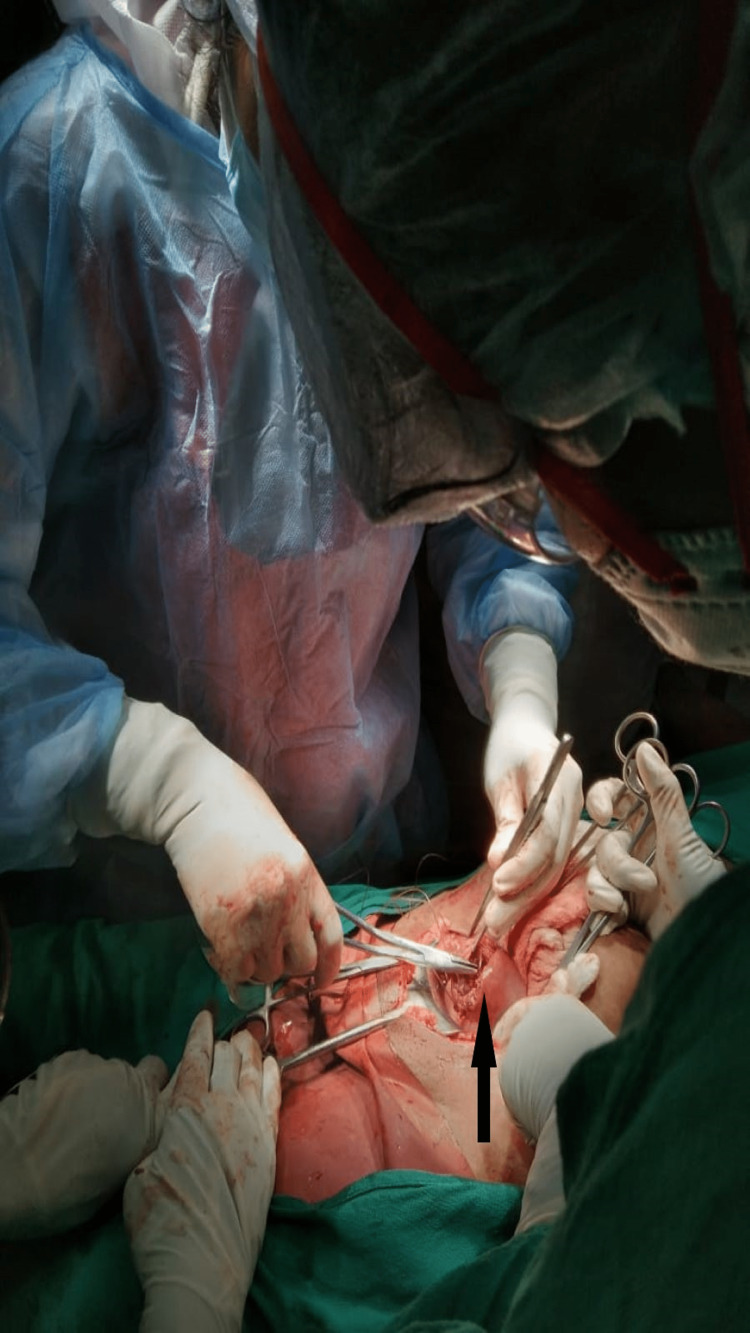
Uterus wound repaired in layers The arrow shows the site of repair

During laparotomy, the abdomen was opened by midline infra-umbilical incision. Approximately one fist of dark old blood clot along with fecal matter necrosed tissue, and fetal tissue was removed from the abdominal cavity without disturbing the bowel loops. The uterus and adjacent structures were explored for the sites of injury. A perforation wound of 2-3 cm in length was seen on the fundus of the uterus likely due to perforation during the procedure of abortion (Figure 1). The Omentum, peritoneum, and parts of the gut were necrosed and gave an eaten-out appearance. Fetal parts were present in the paracolic gutter which were taken out, the whole abdominal cavity was explored and peritoneal toileting with three liters of warm normal saline was done (Figures 2, 3).

The defect of the uterus was repaired in two layers with vicryl 1-0 suture. Necrosed parts of the gut were resected, and anastomosis was done. The abdomen was closed in layers after securing hemostasis. The post-operation patient was shifted to the Intensive care unit where aggressive fluid management with serum electrolytes monitoring was done. She was kept nil per orally for five days, then oral sips of water were allowed followed by a semi-solid diet, her condition gradually improved then she was shifted to the recovery ward after seven days. Thereafter, her recovery was uneventful, and she was discharged after 15 days. Advice for contraception was given.

## Discussion

Targeted history should be obtained from the patient presenting with infection in cases of abortion, as many a time patients try to terminate the pregnancy by using unsafe methods and hesitate to share the information with medical professionals. Sepsis following an abortion is diagnosed clinically when patients present with fever, uterine pain and tenderness, tachycardia, tachypnea, and vaginal bleeding following abortion. Sometimes sepsis progresses rapidly and can be fatal. The presence of fever with a temperature >100.4 ᵒF or hypothermia, tachycardia, respiratory rate >22 breaths per minute, leukocytosis, or leukopenia marks the presence of severe infection. Patients presenting with signs of sepsis syndrome and suspicious of internal organ injury should undergo immediate surgical treatment as soon as they are medically safe for surgery.

Once the patient is recognized as having a case of sepsis following an unsafe abortion broad-spectrum antibiotics, IV fluids and surgical treatment should be given [6-8]. Prevention of unsafe abortion requires a multidisciplinary approach. According to one study by WHO 4.5%-50% of abortions can be reduced by reducing 50% of intimate partner violence [9].

## Conclusions

Various measures need to be taken for reducing the risk of an unsafe abortion such as the prevention of unintended pregnancy by increasing access to contraceptive services and increasing awareness about it. Safe abortion should be accessible to everyone without compromising the privacy of patients. Proper sex education should be given for the prevention of unwanted adolescent pregnancies. In the case of sepsis following a uterine procedure prompt diagnosis and appropriate management with broad-spectrum antibiotics and timely decision for intervention are the only ways to save the patient from an ominous ending as was done in this case.
